# A Canadian naturalistic study of a community-based cohort treated for bipolar disorder

**DOI:** 10.1186/1471-244X-10-24

**Published:** 2010-03-19

**Authors:** Doron Sagman, Bobbie Lee, Ranjith Chandresena, Barry Jones, Elizabeth Brunner

**Affiliations:** 1 Lilly Research Laboratories, Eli Lilly Canada Inc, Toronto, Ontario, Canada; 2 Current address: BYHL, Thornhill, Toronto, Ontario, Canada; 3 Department of Psychiatry, University of Western Ontario, London, Ontario, Canada; 4 Department of Psychiatry, McMaster University, Hamilton, Ontario, Canada

## Abstract

**Background:**

Bipolar illness is associated with significant psychosocial morbidity and health resource utilization. Second generation antipsychotics, used alone or in combination with mood stabilizers are effective in treating acute mania in community settings. This study was designed to compare the change in clinical parameters and resource utilization at one month in a group of patients who required treatment intervention for exacerbation of mania. The clinical response at one year was also evaluated.

**Methods:**

496 patients were enrolled at 75 psychiatric practices across Canada. The Olanzapine cohort (*n *= 287) included patients who had olanzapine added to their medication regimen or the dose of olanzapine increased. The Other cohort (*n *= 209) had a medication other than olanzapine added or the dose adjusted. Changes from baseline in the Young Mania Rating Scale (YMRS), Montgomery Asberg Depression Rating Scale, Beck Anxiety Inventory and SF-12 Health Survey were compared at one month using ANCOVA. Categorical variables at one month for health resource utilization, employment status, abuse/dependency, and the number of suicide attempts were compared using Fisher's Exact test. Patients were followed for one year and a subgroup was evaluated.

**Results:**

At one month, patients in the Olanzapine cohort recorded a mean reduction in the YMRS of 11.5, significantly greater than the mean reduction in the Other cohort of 9.7 (ANCOVA *P *= 0.002). The Olanzapine cohort was significantly improved compared to the Other cohort on the scales for depression and anxiety and did not experience the deterioration in physical functioning seen in the Other cohort. No significant differences were detected in health-related quality-of-life measures, employment status, drug abuse/dependency, number of suicide attempts, mental functioning, emergency room visits or inpatient psychiatric hospitalizations. In a subgroup treated for 12 months with a single second generation antipsychotic, improvements in illness severity measures were maintained with no evidence of significant differences among the antipsychotics.

**Conclusions:**

Patients with bipolar disorder requiring treatment intervention for exacerbation of mania in the community setting responded to olanzapine at one month. In a subset analysis, second generation antipsychotic treatment continued to be beneficial in reducing bipolar symptoms at one year.

## Background

Bipolar disorder is a chronic lifelong illness with many individuals experiencing a relapsing and remitting course. In patients treated for bipolar disorder, relapse rates range from 40% to 60%, making intervention for exacerbation a common necessity[1]. Bipolar illness is associated with significant psychosocial morbidity[2], impacting employment and health resource utilization.

In Canada, the recommended first-line pharmacological treatment for symptoms of acute mania in patients already on therapy is the addition of lithium or divalproex, or a second generation antipsychotic (SGA), or a combination of the two[3]. For non-responsive or relapsing patients already on a SGA, switching to a different SGA or adding lithium are recommended[3]. This study focused on the use of the SGA olanzapine which has been shown to be effective in patients who failed to respond adequately to lithium or valproate monotherapy[4]. SGAs (including olanzapine, risperidone and quetiapine) alone or in combination with mood stabilizers have been reported to be effective in acute mania[3-5].

Clinical trials that demonstrate efficacy and safety for drug approval are randomized and controlled with rigorous patient inclusion criteria. They typically exclude patients with comorbid conditions, substance abuse or concurrent therapy. These studies may not fully reflect the population that ultimately receives drugs in community-based clinical practice[6]. Observational studies can give valid results in real-world clinical settings and can expand the generalizability of results derived from controlled clinical trials[6,7].

The CNS-Bipolar (Canadian Naturalistic Study -- Bipolar Disorder) was designed as a non-interventional, naturalistic, observational study of health outcomes in community-based patients with bipolar disorder receiving routine psychiatric care. Specifically, the study was designed to provide information not often found in randomized clinical trials including: i) one-month outcomes of Canadian patients with bipolar disorder treated in a community-based setting, ii) outcomes in patients with substance abuse and other comorbid conditions, iii) one-year outcomes of patients with bipolar disorder treated under naturalistic conditions in the community, iv) concomitant medications that may be prescribed to patients with bipolar disorder, and v) the impact of treatment on inpatient hospital days and emergency room visits.

The primary objective of CNS-Bipolar was to measure the change in mania symptoms from baseline to approximately one month to capture the response to treatment intervention for an exacerbation of mania. Two cohorts were followed: those in the Olanzapine cohort were prescribed an increase in dose or addition of olanzapine and those in the Other cohort were prescribed a change in dose or addition of a psychotropic medication other than olanzapine. The Young Mania Rating Scale (YMRS)[8] was used to measure the symptoms of mania as it is a valid[8] and well recognized tool[9]. The secondary objectives included measuring response to therapy at one month, degrees of anxiety and depression, health-related quality of life, health resource utilization, and the use of concomitant medications. Patients were then followed for one year and treated as clinically required in an outpatient community setting.

This article describes the statistical comparison of the cohorts at one month. Outcomes at one year are presented for the study group as a whole.

## Methods

The study was a one-year, prospective, observational study of the clinical and health outcomes of community-based outpatients with bipolar disorder who required an adjustment in medication due to exacerbation of mania. The study collected data from 75 psychiatrists practicing in community settings across nine Canadian provinces between April 2003 and March 2005. It was non-interventional in that psychiatrists' decisions regarding treatment were made in the course of routine clinical care and clinic visits every six months. Prior to study initiation, appropriate local ethics committee approval and written informed consent from each patient were obtained. The study was conducted in accordance with the principles of the Declaration of Helsinki[10].

Two treatment cohorts were evaluated. The Olanzapine cohort included patients whose psychiatrist had made the decision to increase the dose of olanzapine or add olanzapine in response to exacerbation of manic symptoms. The Other cohort included patients whose psychiatrist had made the decision to change the dose of a current medication prescribed for bipolar disorder or add another medication (other than olanzapine) in response to exacerbation of manic symptoms. Participating psychiatrists were requested to enroll approximately 60% into the Olanzapine cohort and 40% into the Other cohort, although the final ratio of patients was left to the psychiatrists' discretion (i.e., all or none of an investigator's patients could potentially be enrolled into the Olanzapine or Other cohort). All decisions on medication were made in the course of normal, ongoing patient care and no inducements that might influence prescription choice were provided.

Patients were eligible for the study if they met all of the following criteria: a) presented with bipolar disorder based on Diagnostic and Statistical Manual Version IV (DSM IV) criteria[11]; b) were being treated by a psychiatrist in a community-based office setting; c) were aged 18 years or older; d) were considered by their psychiatrist to be demonstrating manic symptoms and as a result required a change in their ongoing therapy and; e) were capable of providing informed consent for study participation. Patients were excluded from the study if they were receiving olanzapine therapy for bipolar disorder and no dose adjustment of olanzapine was required, or presented for treatment in a hospital psychiatric emergency setting or were considered urgent candidates for hospitalization.

To evaluate treatment outcomes, data was collected at time of medication change (baseline), four to six weeks post baseline (One Month), 22 to 30 weeks post baseline (Six Months), and 48 to 56 weeks post baseline (One Year) during the course of routine psychiatric visits.

Clinical outcomes were evaluated by measuring reduction in illness severity and psychopathology with the YMRS[8], the Montgomery Asberg Depression Rating Scale (MADRS)[12], and the patient-completed Beck Anxiety Inventory (BAI)[13]. A priori, treatment response for a patient was defined as a YMRS score ≤ 8 (from a baseline score of ≥ 9) and MADRS score ≤ 14 at one month. Improvements in health-related outcomes were evaluated with the patient-completed SF-12 Health Survey (the SF-12 is an abbreviated version of the SF-36 Health Survey)[14] which assesses patients' health-related quality of life using physical and mental composite scores and has been shown to be a valid measure in bipolar disorder[15].

Data was also collected on demographic variables, current comorbid conditions, work status, number of suicide attempts, psychiatric hospitalizations, use of psychiatric emergency services, concomitant medications and substance abuse/dependency. Investigators were required to report any serious adverse events for patients taking olanzapine, which were defined as events resulting in death, inpatient hospitalization or prolonged hospitalization, life-threatening events or those causing severe or permanent disability. Nonserious adverse events were not collected.

### Statistical analysis

The study was planned with 87% power to detect a difference in YMRS mean of 3.0 at one month (assuming an SD of 10.0) with 270 patients in the Olanzapine cohort and 180 patients in the Other cohort. Total scores were calculated for the YMRS (primary outcome variable), MADRS and BAI. The SF-12 physical (PCS-12) and mental (MCS-12) composite scores were derived and standardized to a mean of 50 and an SD of 10 in the 1998 general US population[14]. If one or more items of a scale were missing, the total score was considered missing. By a priori definition, only patients with baseline YMRS ≥ 9 were evaluated for potential response. For these patients, response was defined as a YMRS score ≤ 8 and MADRS score ≤ 14 at one month.

Baseline comparisons between the two cohorts were performed using Fisher's Exact tests for categorical variables and ANOVA models with therapy group and site as terms for the dependent variables age, weight and the YMRS, MADRS, BAI, PCS-12 and MCS-12 totals. Sites with less than five subjects were pooled by region.

For the clinical (YMRS, MADRS, BAI) and health survey (PCS-12, MCS-12) outcomes, least-squares means and 95% confidence intervals were generated at baseline and at one month from ANOVA models with therapy cohort and site (pooled) as terms.

Changes from baseline to one month in the clinical and health survey outcomes were calculated for all patients with relevant data. Differences in the change scores between the Olanzapine and Other cohorts were tested using an ANCOVA model with the change score as the dependent variable and therapy group, site (pooled), and baseline value as the independent variables. Least-squares means and 95% confidence intervals were generated from the ANCOVA models for change and for the between therapy group difference in change. Change in weight over the first month was analyzed in the same way as change in the clinical and health survey outcomes. Categorical variables at one month were compared between therapy cohorts using Fisher's Exact test.

Befitting an observational study, physicians were free to modify drug regimens as they felt appropriate after baseline and could add to or drop from either cohort any of the prescribed psychotropic medications. Due to this, comparisons of the two cohorts after one year may not be meaningful. As an exploratory investigation, patients who initiated or changed to olanzapine, quetiapine or risperidone at baseline, and who also completed the full 12 months of treatment with the single SGA assigned at baseline, were selected as a subgroup. Propensity adjusted[16] ANCOVA models controlling for investigative site and baseline levels of the outcome, were used to compare YMRS, MADRS and BAI in the three drug groups. Least-squares means and 95% confidence intervals for change from baseline were calculated. The propensity scores used in the ANCOVA model were the conditional probabilities of being treated with each of the drugs given the individual's baseline covariates, and were obtained from a polytomous logistic model for treatment cohort using as predictors: baseline patient characteristics, health status indicators, utilization of psychiatric and emergency services, and drug and alcohol dependence or abuse indicators.

## Results

**Figure 1:**
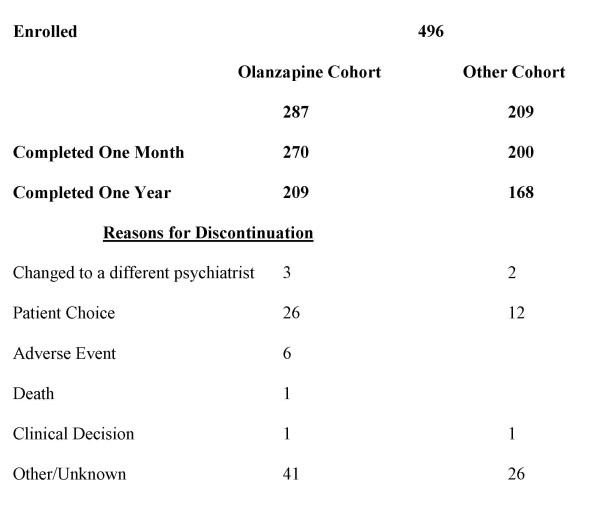
**Patient disposition**.

A total of 496 patients were enrolled into the study, 287 patients in the Olanzapine cohort and 209 patients in the Other cohort (Figure 1).

Demographic and baseline characteristics are shown in Table 1. Subjects were in their early 40s on average, mostly Caucasian, weighed about 80 kg on average and more than half were female. The cohorts were very similar on all measures of bipolar illness severity and use of psychiatric resources (Table 1). The most common comorbid conditions (reported in >7%) were hypothyroidism, hypertension and diabetes, with 35% (101/287) in the Olanzapine cohort and 40% (84/209) in the Other cohort reporting at least one medical condition in addition to bipolar disorder.

**Table 1:** Baseline characteristics of enrolled patients

Characteristics	Olanzapine (*n *= 287)	Other (*n *= 209)	*P *value
Female	55%	67%	0.009
Caucasian	93%	88%	0.038
Mean age, years (SD)	41.7 (12.0)	40.2 (11.5)	0.884
Mean weight, kg (SD)	79.4 (17.3)	82.8 (21.3)	0.048
Smoking status, Yes	47%	41%	
At least one comorbid medical condition	35%	40%	
Work status:			
- employed	38%	36%	
- missed work days	47%	36%	

Past suicide attempts, Yes	39%	35%	

**Baseline Illness Severity LS mean (95% CI)**			

YMRS Total	19.0 (18.2 to 19.8)	19.4 (18.4 to 20.4)	0.524
MADRS Total	14.4 (13.5 to 15.3)	13.5 (12.4 to 14.6)	0.186
BAI Total	14.8 (13.4 to 16.2)	15.3 (13.6 to 17.0)	0.658
SF-12:			
- Physical Composite	50.6 (49.2 to 51.9)	50.5 (48.8 to 52.1)	0.930
- Mental Composite	38.5 (37.0 to 39.9)	37.0 (35.3 to 38.7)	0.189

**Psychiatric Hospitalizations and Use of Psychiatric Emergency Services** **Patients Reporting Use in Last 6 Months**			

Psychiatric Emergency Room Visits	20%	20%	
Inpatient Admissions	21%	21%	
Lifetime Inpatient Admissions	65%	61%	

YMRS: Young Mania Rating Scale

MADRS: Montgomery Asberg Depression Rating Scale

BAI: Beck Anxiety Inventory

At baseline, 38% (107/283) in the Olanzapine cohort and 36% (74/205) in the Other cohort were employed (Table 1), with 47% (47/101) in the Olanzapine cohort and 36% (24/66) in the Other cohort reporting missed work days.

### Clinical results at one month

At one month, 94% (*n *= 270) of patients in the Olanzapine cohort and 96% (*n *= 200) of patients in the Other cohort remained in the study.

**Figure 2:**
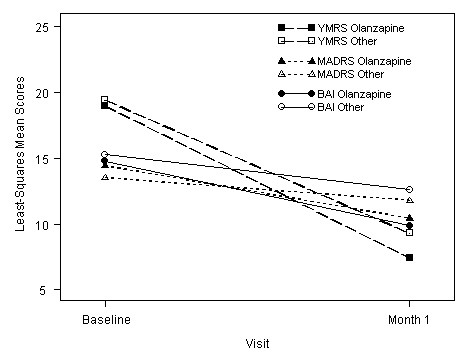
**Least-squares mean scores for YMRS, MADRS, and BAI at baseline and one month**. YMRS: Young Mania Rating Scale. MADRS: Montgomery Asberg Depression Rating Scale. BAI: Beck Anxiety Inventory.

The primary outcome measure was the change in the YMRS score from baseline to one month. In the Olanzapine cohort a mean reduction in the YMRS of 11.5 (95%CI -12.2 to -10.7) was recorded at one month. The change represented a reduction from a mean YMRS at baseline of 19.0 (95%CI 18.2 to 19.8) to a mean at one month of 7.4 (95%CI 6.7 to 8.2) (Figure 2). In the Other cohort a mean reduction of 9.7 was recorded (95%CI -10.6 to -8.8) representing a reduction from a mean of 19.4 (95%CI 18.4 to 20.4) to a mean of 9.3 (95%CI 8.4 to 10.2) (Figure 2). The reduction in mean YMRS from baseline in the Olanzapine cohort was significantly greater compared to the Other cohort with a difference of -1.8 (95%CI -2.9 to -0.6, ANCOVA, *P *= 0.002).

In the Olanzapine cohort 44% (95%CI 37.3 to 50.3) met the a priori criterion for response, significantly more than the 34% (95%CI 26.5 to 40.6) in the Other cohort (Fisher's Exact test, *P *= 0.049).

Figure 2 shows the reductions in mean scores on the YMRS, MADRS and BAI for both cohorts at one month. On the MADRS, a mean reduction of 3.9 (95%CI -5.0 to -2.9) was found in the Olanzapine cohort at one month, while in the Other cohort a mean reduction of 2.3 (95%CI -3.5 to -1.1) was found (ANCOVA, *P *= 0.031). On the BAI the Olanzapine cohort recorded a mean reduction of 4.9 (95%CI -6.1 to -3.7) at one month while in the Other cohort a mean reduction of 2.6 (95%CI -4.0 to -1.2) was found (ANCOVA, *P *= 0.012).

### Health outcomes at one month

A reduction on the physical composite scale of the SF-12 was seen in the Other cohort at one month, reflecting deterioration, which was not seen in the Olanzapine cohort (mean reduction in Olanzapine cohort 0.0, 95%CI -1.1 to 1.1, mean reduction in Other cohort 1.7, 95%CI -3.0 to -0.5, ANCOVA *P *= 0.036). On the mental composite scale of the SF-12, improvements were recorded for both cohorts at one month, although the change from baseline did not differ significantly between the groups (mean improvement in Olanzapine cohort 3.4, 95%CI 2.0 to 4.9, mean improvement in Other cohort 2.1, 95%CI 0.5 to 3.8, ANCOVA *P *= 0.236). At one month, 1.5% (4/267) in the Olanzapine cohort and 1.5% (3/197) in the Other cohort had made at least one suicide attempt in the past 30 days.

At one month, no differences in the employment measures were detected between the groups with 34% (90/268) in the Olanzapine cohort and 34% (64/191) in the Other cohort employed and 36% (20/56) in the Olanzapine cohort and 30% (14/46) in the Other cohort reporting missed work days.

In the first month, 4% (12/268) in the Olanzapine cohort required inpatient psychiatric hospitalization, not significantly different from the 6% (11/198) in the Other cohort. The number requiring psychiatric emergency room visits was also similar; 3% (9/267) in the Olanzapine cohort and 4% (8/197) in the Other cohort.

No obvious differences were found in the number of patients abusing alcohol and/or cannabis at one month (alcohol abuse in Olanzapine cohort 11%, 27/253, in Other cohort 8%, 15/193: cannabis abuse in Olanzapine cohort 6%, 14/252, in Other cohort 5%, 9/191).

Patients in both cohorts gained weight by one month, but the increase in weight did not significantly differ between the two groups (ANCOVA *P *= 0.5215). The Olanzapine cohort gained a mean of 1.2 kg (95%CI 0.3 to 2.1) while the Other cohort gained a mean of 1.7 kg (95%CI 0.6 to 2.8).

### Treatment patterns at one month

SGA use remained stable over the first month in both groups. At one month 245 were taking olanzapine (86%, 232/270 in Olanzapine cohort and 7%, 13/200 in Other cohort), 62 were taking quetiapine (1%, 4/270 in Olanzapine cohort and 29%, 58/200 in other cohort) and 81 were taking risperidone (1%, 3/270 in Olanzapine cohort and 39%, 78/200 in Other cohort).

Table 2 captures the use of SGAs, mood stabilizers and selective serotonin reuptake inhibitors/selective serotonin and norepinephrine reuptake inhibitors (SSRIs/SNRIs). The pattern of psychotropic drug use for the combinations captured is similar, with the combination of a SGA and mood stabilizer being the most common (used by 33%, 90/270 in the Olanzapine cohort and 30%, 60/200 in the Other cohort at one month).

**Table 2:** Use of second generation antipsychotics, mood stabilizers and SSRI/SNRIs^†^

Medication*	Baseline		One Month	
	**Olanzapine** **(*n *= 287)**	**Other** **(*n *= 209)**	**Olanzapine** **(*n *= 270)**	**Other** **(*n *= 200)**

**SGA + Mood** **Stabilizer**	97 (34%)	55 (26%)	90 (33%)	60 (30%)

**SGA Alone**	87 (30%)	42 (20%)	75 (28%)	37 (19%)

**SGA + SSRI/SNRI**	51 (18%)	23 (11%)	43 (16%)	30 (15%)

**SGA + SSRI/SNRI +** **Mood Stabilizer**	34 (12%)	24 (11%)	39 (14%)	27 (14%)

**Mood Stabilizer Alone**	8 (3%)	38 (18%)	7 (3%)	28 (14%)

**Mood Stabilizer + SSRI/SNRI**	5 (2%)	13 (6%)	6 (2%)	8 (4%)

**SSRI/SNRI Alone**	3 (1%)	8 (4%)	3 (1%)	3 (2%)

SGA: second generation antipsychotic

^†^SSRI/SNRIs: selective serotonin reuptake inhibitors/selective serotonin and norepinephrine reuptake inhibitors: includes citalopram, fluoxetine, paroxetine and venlafaxine

Mood Stabilizer: includes lithium and valproate

*In addition patients may have used benzodiazepines and/or tricyclics

### Outcomes at one year

Over the course of one year, psychiatrists were free to alter psychotropic medication as clinically required, resulting in mixed medication profiles for both groups. For this reason one-year results are not presented by the treatment cohorts defined for the one-month analysis. A total of 377 patients completed the one-year study. Patients discontinued for the reasons summarized in Figure 1.

One patient taking olanzapine died following the one-month visit but before the six-month visit (Figure 1), although the death was deemed by the investigator to be unrelated to olanzapine. Twenty-one patients taking olanzapine were hospitalized for adverse events: six for depression, six for bipolar disorder, three for suicidal ideation/attempt, two for mania, one for hypomania, one for psychosis, one for agitation, and one for hyponatremia. One relapse of depression was deemed possibly related to olanzapine, while all other events were considered not drug related. Psychiatrists were not required to report hospitalizations or other serious events for patients taking SGAs other than olanzapine. Over the course of the year, six patients discontinued due to adverse events (Figure 1).

Changes in the YMRS, MADRS and BAI between baseline and one year were analyzed by single SGA used (patients may have used medications other than an SGA, but those taking combination SGAs were excluded). An adjustment using the propensity method for the probability of being assigned a specific single SGA was effective in reducing bias due to baseline variables. While treatment with a single SGA was associated with improvements on the YMRS, MADRS and BAI at one year, there were no significant differences in the degree of improvement among patients treated with olanzapine, risperidone and quetiapine (Table 3). For the SF-12, treatment with a single SGA was associated with improvements on the mental but not the physical composite score at one year, with no significant differences in the degree of improvement among patients treated with olanzapine, risperidone and quetiapine.

**Table 3:** Mean change in YMRS, MADRS, BAI and SF-12 at one year by single SGA received

Single SGA Received	LS Mean for Change from Baseline to One Year with Propensity Adjustment	95% Confidence Interval	*P *value*
**YMRS**			

olanzapine (*n *= 154)	-15.9	-16.7 to -15.2	0.835
	
risperidone (*n *= 41)	-15.9	-17.3 to -14.5	
	
quetiapine (*n *= 38)	-15.5	-16.9 to -14.1	

**MADRS**			

olanzapine (*n *= 154)	-7.5	-8.8 to -6.2	0.552
	
risperidone (*n *= 42)	-6.2	-8.7 to -3.7	
	
quetiapine (*n *= 37)	-7.8	-10.4 to -5.3	

**BAI**			

olanzapine (*n *= 132)	-7.0	-8.4 to -5.6	0.935
	
risperidone (*n *= 34)	-6.9	-9.7 to -4.1	
	
quetiapine (*n *= 32)	-6.4	-9.4 to -3.5	

**SF-12 Mental Composite**			

olanzapine (*n *= 127)	6.3	4.0 to 8.7	

risperidone (*n *= 37)	5.0	0.5 to 9.5	0.057
	
quetiapine (*n *= 31)	11.9	7.1 to 16.8	

**SF-12 Physical Composite**			

olanzapine (*n *= 127)	0.6	-1.4 to 2.5	0.077
	
risperidone (*n *= 37)	-0.7	-4.4 to 3.0	
	
quetiapine (*n *= 31)	-4.4	-8.4 to -0.5	

SGA: second generation antipsychotic

YMRS: Young Mania Rating Scale

MADRS: Montgomery Asberg Depression Rating Scale

BAI: Beck Anxiety Inventory

*P value from F-test for any differences among the effects of the three single SGAs

## Discussion

The overall purpose of CNS-Bipolar was to study outcomes at one month in bipolar disorder following a change in pharmacological treatment for exacerbation of mania in the outpatient psychiatric setting. Community-based settings capture all presenting patients, regardless of comorbid conditions -- in this study 35 to 40% of the patients enrolled had at least one comorbid medical condition. The heterogeneity of previous history, treatment protocols and investigators will all influence outcomes. Clinically meaningful reductions in YMRS scores were observed in both treatment groups at one month (11.5 for the Olanzapine cohort and 9.7 for the Other cohort). The absolute YMRS scores at one month were 7.4 for the Olanzapine cohort and 9.3 for the Other cohort. YMRS scores below 12 are generally considered a clinical indicator of remission[4,17]. Patients in the Olanzapine cohort recorded a significantly greater mean reduction in the YMRS of 11.5 compared to the Other cohort (mean reduction of 9.7), and significantly more patients in the Olanzapine cohort responded to treatment (44%) compared to the Other cohort (34%).

In this study, psychiatrists were free to modify drug regimens as they felt appropriate and could add olanzapine to regimens of the Other cohort or add another SGA to regimens of the Olanzapine cohort. A SGA and mood stabilizer was the most common drug combination used at one month (30 to 33% of patients) and consistent with the clinical practice guidelines for the treatment of acute mania[3]. Tohen documented a YMRS reduction of 13.1 at six weeks when olanzapine was added to lithium or valproate compared to a reduction of 9.1 on mood stabilizers alone[4]. A literature review[18] and meta-analyses[17,19] have found olanzapine cotherapy to be superior to monotherapy on measures of treatment response, change in YMRS, and treatment withdrawal.

In addition to improvements in mania symptoms, improvements in depression and anxiety were reflected in both cohorts on the MADRS and BAI. Improvements in the Olanzapine cohort were significantly greater than the improvements recorded in the Other cohort on both measures. The improvement was consistent with changes recorded in the Hamilton Depression Rating Scale at six weeks in patients taking combined olanzapine and a mood stabilizer[4,20]. On the other hand, a review of six olanzapine trials[9] found olanzapine to be no more efficacious than divalproex in reducing depressive symptoms.

According to the physical component of the SF-12, the Other cohort demonstrated deterioration in the first month; this was not seen in the Olanzapine cohort. In contrast, Namjoshi found that olanzapine treatment alone[21,22] or combined with a mood stabilizer[20] improved physical functioning in acute mania. Patients in both treatment cohorts experienced improvement in the mental component score on the SF-12 but there was no significant between-group difference in the degree of improvement. Our study results may reflect a pattern identified by Namjoshi[21] who found that symptom reduction (reflected in a 10-point reduction in the YMRS) can occur within three weeks with olanzapine treatment, while improvements on health-related quality-of-life measures take longer to achieve. After reviewing the quality-of-life literature in bipolar disorder, Michalak[15] concurred -- clinical symptoms and measures of physical functioning could improve with acute treatment while other quality-of-life measures could take up to a year to register an improvement.

This pattern may explain the failure to detect at one month any meaningful changes in employment status or number of missed work days, emergency room visits, inpatient hospitalization, suicide attempts, or the use/abuse of cigarettes, alcohol or cannabis. Others have recorded improvements in work status after 12 weeks of olanzapine treatment[22].

Weight gain of 1 to 2 kg was recorded in both treatment groups at one month but did not significantly differ between the groups. The gain appears to be less than that reported in studies that used olanzapine in combination with mood stabilizers (2.9 kg[19], 3.1 kg[4]), and similar to the gains when olanzapine was used as monotherapy (2.1 kg gain found in a four-week olanzapine vs. placebo controlled clinical trial[23], 1.65 kg gain in three placebo-controlled monotherapy trials[9])

Retention rates were high in this study and consistent with rates reported in a review of controlled olanzapine trials[9], although others have reported high withdrawal rates in the bipolar population[19]. Over 75% of patients remained on therapy for one year which at least in part may be attributable to patient and investigator perceived clinical improvements in manic, depressive and anxiety symptoms and the flexibility psychiatrists had to alter medication as required.

Following a year of community-based treatment, it was only possible to consider outcomes by treatment received and patients were grouped by single SGA used (those receiving multiple SGAs were excluded). In this exploratory subgroup analysis the improvements in bipolar symptoms seen in these patients at one month were maintained at one year and there were no significant differences among patients treated with olanzapine, risperidone or quetiapine. These results are consistent with a meta-analyses of SGA monotherapy which found no significant differences between SGAs[17].

Similarly, significant differences were not seen in the health-related quality-of-life outcomes between olanzapine, risperidone or quetiapine when used as a single SGA over one year. In keeping with the one-month results, mental functioning was improved but an improvement was not seen in physical functioning. These results fail to replicate those of Namjoshi who found improvements in quality-of-life measures following one year of open-label olanzapine treatment[21].

There were a number of limitations presented by this study design. Patients in the Other cohort may have received olanzapine and patients in the Olanzapine cohort may have received other SGAs. At one month the degree of mixing of medication was small. At one year patients were grouped by monotherapy used; however, the study had low power for this exploratory analysis. The primary efficacy measure (YMRS) was scored by the investigator. Inherent in observer rated open-label studies is the risk of performance bias. Another limitation was that the reporting of nonserious adverse events was at the discretion of the investigator and was not mandatory in this observational study. Serious adverse events were only reported for those in either cohort taking olanzapine.

## Conclusions

CNS-Bipolar involved a large sample of community-based patients with a diagnosis of bipolar disorder experiencing an exacerbation of manic symptoms. It had broad inclusion criteria and enrolled patients with comorbid conditions, substance abuse/dependency and a wide variety of concomitant medications. Patients who were prescribed olanzapine or required a change in dose of olanzapine demonstrated significantly greater improvements in manic, depressive and anxiety symptoms at one month compared to others who received different therapeutic intervention for mania. At one month no prominent improvements in health-related quality-of-life measures were observed and the number of suicide attempts, rate of alcohol and cannabis use and the use of health care resources were similar in the Olanzapine cohort and Other cohort. At one year the improvements in bipolar symptoms were maintained, although there was no association with the type of SGA used.

The results suggest that adjunctive olanzapine may be clinically beneficial in the community setting for the treatment of acute mania, with improvements in bipolar symptoms detected at one month. In a subset analysis, SGA treatment continued to be beneficial in reducing bipolar symptoms at one year. Intervention that achieves fast, measurable and lasting results on symptoms of mania, depression and anxiety is of clinical advantage in bipolar patients.

## Competing interests

Doron Sagman: The author is employed by Eli Lilly Canada and holds company shares, but will not gain financially from the publication of this manuscript, now or in the future. The author will be reimbursed by Eli Lilly Canada for the article processing charge.

Bobbie Lee: The author was employed by Eli Lilly Canada when the analyses were conducted and the manuscript prepared, but will not gain financially from the publication of this manuscript, now or in the future. The author reviewed and approved the final manuscript at her new institution, BYHL, Thornhill, Ontario, Canada. The author still holds company shares.

Ranjith Chandrasena: The author was an investigator in the study and was compensated for his participation.

Barry Jones: The author was employed by Eli Lilly Canada at the time of protocol development but will not gain financially from the publication of this manuscript, now or in the future.

Elizabeth Brunner: The author is employed by Eli Lilly and Company and holds company shares, but will not gain financially from the publication of this manuscript, now or in the future.

## Authors' contributions

DS made substantial contributions to interpretation of the data and preparation of the manuscript. BL made substantial contributions to the design of the study and the statistical analysis of the data. RC participated in the acquisition of the data and was involved in revising the manuscript. BJ made substantial contributions to conception and design of the study and the interpretation of the data. EB made substantial contributions to conception and design of the study and the interpretation of the data. All authors read and approved the final manuscript.

## Pre-publication history

The pre-publication history for this paper can be accessed here:

http://www.biomedcentral.com/1471-244X/10/24/prepub
